# Does the principle of investment diversification apply to the starting pitching staffs of major league baseball teams?

**DOI:** 10.1371/journal.pone.0244941

**Published:** 2021-01-13

**Authors:** Paul J. Roebber

**Affiliations:** Atmospheric Science Program, Department of Mathematical Science, University of Wisconsin–Milwaukee, Milwaukee, Wisconsin, United States of America; Vilnius University, LITHUANIA

## Abstract

Financial advisors often emphasize asset diversification as a means of limiting losses from investments that perform unexpectedly poorly over a particular time period. One might expect that this perceived wisdom could apply in another high stakes arena–professional baseball–where player salaries comprise a substantial portion of a team’s operational costs, year-to-year player performance is highly variable, and injuries can occur at any time. These attributes are particularly true in the case of the starting pitching staffs of professional baseball teams. Accordingly, this study analyzes starting pitcher performance and financial data from all Major League Baseball teams for the period 1985–2016 to determine whether the standard investment advice is applicable in this context, understanding that the time horizon for success for an investor and a baseball team may be distinct. A multiple logistic regression model of playoff qualification probability, based on realized pitcher performance, measures of luck, and starting pitcher staff salary diversification is used to address this question. A further stratification is conducted to determine whether there are differences in strategy for teams with allocated financial resources that are above or below league average. We find that teams with above average resources increase their post-season qualification probability by focusing their salary funds on a relative few starting pitchers rather than diversifying that investment across the staff. Second, we find that pitcher performance must align with that investment in order for the team to have a high qualification probability. Third, the influence of luck is not negligible, but those teams that allocate more overall funds to their pitching are more resilient to bad luck. Thus, poorly resourced teams, who are generally unable to bid for pitchers at the highest salary levels, must adopt alternative strategies to maintain their competitiveness.

## Introduction

In finance, one hears of the importance of a diversified investment portfolio. Diversification in this context entails spreading investment dollars in ways that are minimally correlated, so that market moves in a particular sector will not risk the overall portfolio. Typically, this spreading is accomplished by investing in a mix of assets such as stocks (placed in shares across different sectors), real estate, cash and fixed interest vehicles such as bonds. Put more simply, by spreading a (presumably) fixed amount of investment dollars, one insures that these funds are not concentrated in the worst performing assets. This strategy, of course, also insures that returns will be less than that obtainable from the very best performers in that same portfolio, a tacit admission that the predictability of future performance of any individual asset in this context is low.

The analogy to professional baseball teams is as follows. The most valuable major league baseball (MLB) franchises were worth several billion dollars at the beginning of 2020 [1], but ownership necessarily invests considerably less in player payroll ($59-$254 million [2]), and team management must decide specifically how to allocate those funds. Ultimately, but perhaps with some exceptions [3,4], the goal of professional teams is to win games and in so doing, to win championships.

It is self-evident that in order to win games, teams must do their best to score runs while limiting the number of runs scored by their opponents. Over a MLB season consisting of 162 games, runs scored and runs allowed correlate very highly with the won-lost record of those teams, and is encapsulated by what has become known as the “Pythagorean expectation” formula [5,6], expressed as:

$$win\;frequency=\frac{{RS}^{a}}{{RS}^{a}+{RA}^{a}}$$ (1)

where *RS* is the team’s runs scored, *RA* is the team’s runs allowed and *a* is an exponent (variously ranging from 1.8 to 2). Analysis of this formula shows that in modern MLB scoring contexts, an increment of 10 runs (scored or saved) is worth approximately one additional win over a 162 game season.

Thus, the challenge is to decide where to allocate available funds in order to obtain the talent necessary to score runs and/or prevent run scoring by the opposition. Team wins above replacement (WAR) level data [7,8], obtained from BaseballReference.com for the period from 1901-present (considered to be modern MLB history) shows that teams with a season winning percentage of 0.600 or greater (a level that for the modern playoff format will typically assure a post-season berth) obtained ~60% of this win value from offense and the remainder from pitching and defense. We note, however, that there has been considerable variation in the emphasis that successful teams have placed on pitching and defense, which has ranged as high as 66% in recent years (the 1995 Atlanta Braves). Emphasizing this important role is the fact that pitchers dominate the list of top MLB earners [2]. One complication to investment is the understanding that MLB pitchers are considerably more at risk of injury than regular position players (from 1989–1999, 48.4% of all MLB disabled list reports involved pitchers [9] and this circumstance has continued in more recent years [10]. Further, this disproportionate rate of injury is also reflected in financial impact (e.g., in the 2015 season, ~59% of total salary spent on players on the disabled list was accounted for by pitchers, with second base and catcher tied for the second-highest percentage at less than 6% [11].

Owing to these factors, we choose here to focus on the role of pitchers in our analysis of MLB team talent diversification. We focus in particular on the 5 pitchers on a team who have faced the most batters in a given season, using performance and salary data for the 1985–2016 MLB seasons. We note that for the most part, this stratification will encompass the so-called starting pitching staff, those pitchers who begin the game and who will pitch on average for at least 6 innings in a 9 inning game. For the aforementioned 1995 Atlanta Braves, for example, this set of pitchers, ordered by batters faced, was Tom Glavine, John Smoltz, Greg Maddux, Steve Avery and Kent Mercker, a group that started all of that year’s games for that team and were responsible for 70% of all the batters their team’s pitchers faced.

In the following sections, we will provide information on the data used for the analysis, the specific outcome measures, details regarding the diagnostic model and conclusions obtained from that analysis, with the overall objective being to answer the question as to whether or not diversification of salary investment in the starting pitching staff produces more wins over a MLB season.

## Methods

### Available data and measures

The measures used in this study (Table 1) encompass measures of pitcher performance, expected game outcomes from that performance, and pitcher salary data. Performance data for the pitchers for each team for 1985–2016 and team-level data were obtained from Fangraphs.com. All salary and pitcher game Score data (our expected game outcome measure, see below) used for this study were obtained for this same period from BaseballReference.com. Preseason and playoff games were not included in these data, in order to maintain sample size (the regular season is much longer) and competitive context (preseason games are not focused on wins, postseason games involve a higher level of competition and often distinct strategies [12]).

**Table 1:** Measures used.

*Measure*	*Description*
SALn	Team starting pitching staff salary normalized to league (investment)
GSc	Season average pitcher game score (outcome indicator)
FIP	Fielder independent pitching on an earned run average scale (realized performance)
BABIP	Batting average on balls in play (luck factor)
LOB%	Left on base percentage (luck factor)
Gini	Starting staff salary inequality (salary diversification)

There are a number of available measures of pitching effectiveness and pitching outcomes. Here, we will examine: fielder independent pitching on an earned run average scale (FIP), Batting Average on Balls in Play (BABIP), Left On Base percentage (LOB%) and Pitcher Game Score (GSc). We discuss these measures in turn below–further details can be found in the cited references and numerous other sabermetric studies both in the literature and online.

Earned run average (ERA) has long been a measure of choice for evaluating pitcher effectiveness. ERA is simply the rate of *earned runs* allowed by a pitcher in 9 innings (note that runs scored against a pitcher can be deemed “earned”, meaning the responsibility of that pitcher, or “unearned”, meaning the fault of his defense, such as when a runner who reaches base on a fielder error later scores). Unfortunately, there are several factors that confound the interpretation of ERA. First, what is deemed a hit versus an error is subjective and at the whim of the official scorekeeper at a game. While there is some quality control over scorekeeping, this subjective aspect reduces the utility of the portioning between earned and unearned runs. Second, the rate of balls in play that fall in for hits (i.e., BABIP) can fluctuate substantially from one season to the next for a given pitcher [13]. This variation can be simply a matter of luck (a ball can be hit hard but directly at a fielder, whereas another ball may be hit softly but fall in safely), or it may be modified by the quality of the defense working behind the pitcher. It is worth noting that research suggests that pitchers can have some control over the outcomes of balls in play, but this is a lesser effect [14]. FIP [15,16] is an ERA-like measure that attempts to control for the above factors by estimating what a pitcher’s ERA would be had he experienced league average BABIP, using only the outcomes over which he has direct control (bases on balls, hit batters, home runs, and strike outs). Thus, the confounding role of defensive quality and luck is taken out of the calculation for FIP, which is defined as:

$$FIP=\frac{13*HR+3*(W+HP)-2*K}{IP}+constant$$ (2)

where *HR* are home runs allowed, *W* are walks, *HP* are batters hit by the pitch, *K* are strikeouts, and *IP* is innings pitched. The constant is set to align league average FIP with league average ERA in a given season (a typical value, according to Fangraphs, is 3.10).

BABIP measures how often a ball that is hit into play (i.e., a batter plate appearance that ends in something other than a *HR*, *W*, *HP*, *K*, catcher interference, or a sacrifice bunt) results in a hit. As noted above, the variability in this measure for a pitcher from one season to the next is largely, but not entirely, the result of luck and the talent of the defense behind him. The part that the pitcher has some control over is how hard a ball is hit, and so more-talented pitchers may consistently produce lower BABIP than less-talented pitchers. Furthermore, pitchers who produce more fly balls and strike outs tend to have lower BABIP than others. However, the effects of luck and defensive quality are sufficiently large as to produce considerable variation in this value from one season to the next and one generally assumes that over time, a pitcher’s BABIP should regress to near the league average. The calculation at FanGraphs is:

$$BABIP=\frac{H-HR}{AB-HR-K+SF}$$ (3)

where *H* are hits, *AB* are at bats, and *SF* are sacrifice flys.

Left On Base percentage (LOB%) measures how many baserunners the pitcher allowed that did not subsequently score. Here, it is based on season totals rather than individual game box scores, and is calculated by:

$$LOB\%=\frac{H+W+HP-R}{H+W+HP-1.4*HR}$$ (4)

As with BABIP, this exhibits a substantial random element and pitchers tend to regress towards the league average (typically near 72% with plus/minus 10% representing the range from excellent to poor) over time. But also as with BABIP, some pitchers have more control over this measure–for example, strikeout pitchers tend to produce higher values of this measure since a ball not put into play will not allow a runner to advance compared to a ground ball to the right side of the infield or a deep fly ball.

Pitcher Game Score (GSc) is a metric used to judge the quality of a starting pitcher’s performance in a single game [17,18]. Here we aggregate the GSc for all starts in a season and normalize it (in cases where the season was shorter than 162 games) to a 162 game season. An individual GSc is computed from a baseline of 50, which is set as average, with an expected range of 0–100 (with some rare exceptions that are lower or higher). The usefulness of this score is that it effectively identifies games that, under normal conditions, would register a win for the starting pitcher and thus can be used independently from offensive support.

As a check on this assertion, we have analyzed all season average GSc for all MLB starting pitchers from 1985–2016, weighted by their number of decisions (wins plus losses) and compared this to the overall winning percentage of all teams during this period (0.499; note that it is not 0.500 since some decisions are awarded to relief pitchers which we have excluded from this analysis). The weighted average GSc is 49.6, indicating that an average outing should give an average chance of winning, as desired. The GSc for an individual start is computed as:

$$Gsc=50+K-2H-2(R+ER)-W+O+2{\left(IP-4\right)}_{IP\geq 4}$$ (5)

where *ER* are earned runs, *O* are outs, and the final term on the right-hand side is evaluated only if the pitcher has pitched for at least 4 innings. These individual game scores are then averaged over all starts for a given pitcher in a particular season. We further aggregate these individual pitcher season averages to generate a team GSc consisting of the game scores of the 5 pitchers analyzed for each team in a season, weighted by their decisions in that season.

Two financial measures used in this study are normalized staff salary (SALn) and the staff Gini coefficient (Gini). We determine SALn by computing the league average staff salary (that is, the accumulated salaries of the 5 identified pitchers for each team) and the variance of those salaries across the league, and then performing a league normalization:

$$SALn=\frac{Staff\;Salary-League\;Average}{League\;Standard\;Deviation}$$ (6)

where SALn is measured in thousands of dollars. SALn measures how many standard deviations the 5-pitcher staff of a given team in a given season is above or below the league average, and allows us to compare staff salaries across the 32 years of the study, a period over which MLB team staff salary variance increased by nearly a factor of 400.

The Gini coefficient is a standard measure of income or wealth inequality across a population. It scales in the range from 0–1, where 0 is perfect equality and 1 is perfect inequality (i.e., all the wealth is held by one member of the group). Thus, it can be considered a measure of the deviation from perfect equality, and a higher coefficient indicates that higher (here, salaried) individuals receive larger percentages of the total salary of the population. It is computed for staff salary as:

$$Gini=\frac{\sum_{i=1}^{n}\sum_{j=1}^{n}\left|S_{i}-S_{j}\right|}{2n^{2}\bar{S}}$$ (7)

where n is the number of pitchers (here equal to 5), the S_i_ are the individual pitcher salaries, and $\bar{S}$ is the average of the 5 pitcher salaries on that team. In the present study, a small team Gini coefficient signifies diversification of salary dollars across the staff and a large value indicates a concentration of those dollars (i.e., less diversification).

## Results and discussion

In order to understand pitching staff diversification, it is useful to consider the edge case of a team with unlimited resources. This team can afford to spend as much as it needs to in order to obtain expected pitching talent, its only constraint being that prediction of future performance is difficult. In order to determine the impact of this factor, we perform a regression analysis of GSc and SALn (which as described above, are computed at the 5-man starting staff level rather than for individuals). This regression provides the following relationship (at well above the 99% confidence level):

GSc=49.7+1.164*SALn (8)

where the 95% confidence interval for the above regression coefficient is 0.953–1.376. This equation states that, starting at roughly a 0.500 winning percentage (which we previously showed is tied to a GSc of ~50), we can gain 0.953–1.376 GSc points (or linearly, 1.5–2.2 wins over 162 games, but see below) for each standard deviation of staff salary expenditure. In 2016 dollars, this translates to a cost of between $23-$36 million for that standard deviation improvement or a minimum of a 10% salary addition to the 2020 highest team payroll and more than one-third of the total payroll for several teams. Perhaps more prohibitive than this cost, though, is that this spending accounts for only 11% of the variance in GSc, again meaning that expected (as measured by salary) and actual (as measured by GSc) pitching outcomes can diverge substantially.

**Fig 1:**
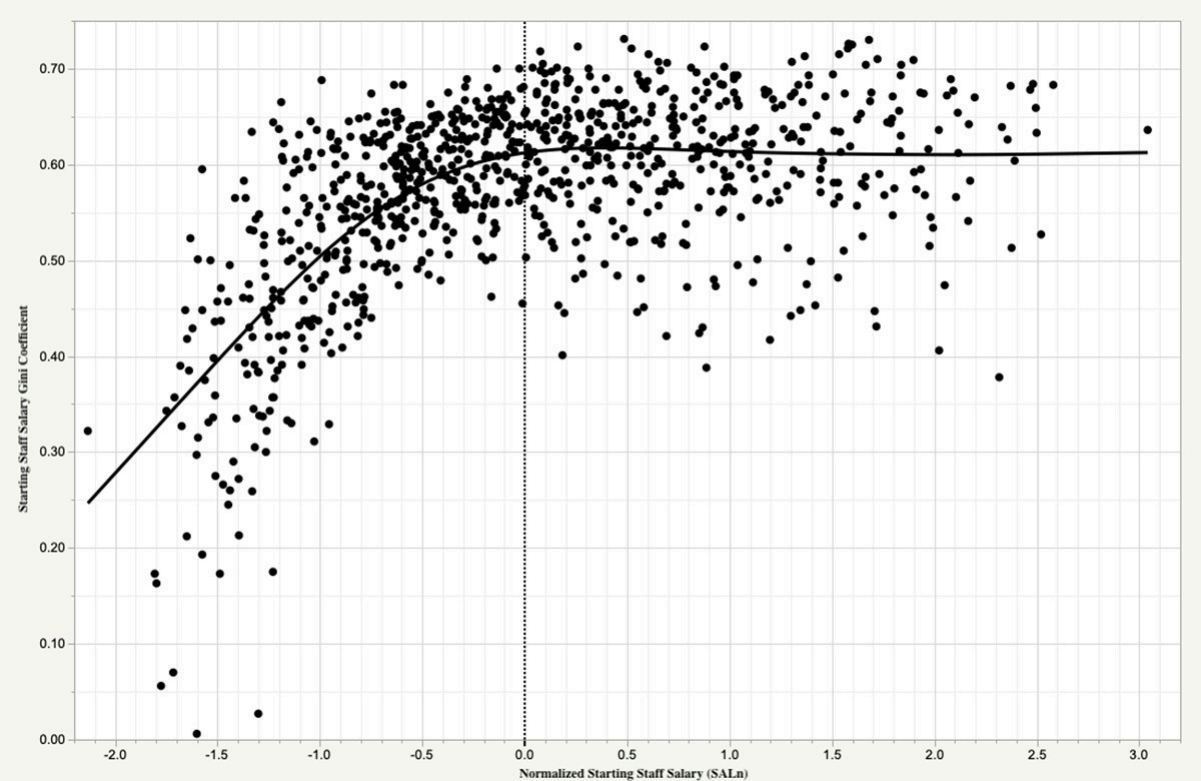
Starting pitching staff salary distributions in MLB 1985–2016. Shown is the relationship between team starting pitching Game Score (GSc) and starting pitching Gini coefficient for all MLB teams for each season 1985–2016. Also shown by the solid line is the smoothed relationship between these quantities.

This result might be an argument to diversify. If the cost of wins is high and is irrevocably attached to outcome uncertainty (owing to performance on the field and to injury), might a team be better off avoiding such bets on one individual? Rather, by spreading funds across several lower cost individuals, it might increase the chance that at least one bet will pay off. The MLB salary market, however, seems to mitigate against this (Fig 1). Evidently, the “star-centered” nature of sports and entertainment leads to less diversification as team resources increase, where in the case of MLB the Gini coefficient asymptotes rapidly to ~0.6 as starting staff salary reach’s league average values. In fact, the median starting staff salary Gini coefficient for the 1985–2016 period is 0.593. For reference, income Gini for countries around the world range from 0.242 (Slovenia) to 0.630 (South Africa), and for the North American continent, 0.338, 0.414, and 0.454 in Canada, the United States, and Mexico, respectively [19].

A second operational concern that might operate against the kind of diversification strategy used in financial markets is that spreading funds inevitably leads to more average results as a consequence of that approach. But unlike with a stock portfolio, the time horizon for a MLB team is short, essentially the current season if it has any hopes of making the playoffs. Since a few wins over 162 games can make the difference of making the post-season or not, what might appear to be a risky, disproportionate investment could be the preferred strategy in this situation.

To answer these questions, we reorient the analysis to the probability of qualifying for the postseason. In the MLB wild card post-season format in place since 2012, this translates to an average minimum season win total of at least 88 games, which yields a median team GSc of 52 in the actual data (recall that actual team wins are derived from offense, pitching, and defense combined, not one facet of the game alone). Note that this result also provides another perspective on the raw salary expenditure discussed above–the one standard deviation investment could more easily move a team to the postseason than a linear analysis would suggest.

In order to gain some understanding of these questions, we build a multiple logistic regression model of playoff qualification probability based on the 88 game/GSc threshold. Notably, logistic regression is well-suited for this task since it predicts a probabiity of an outcome rather than the outcome itself. As a result, the method is widely applied across fields as disparate as medicine, social sciences, political science, engineering, marketing, and economics [20–23].

The inputs to the model are measures of *achieved* performance (FIP), predominant factors of luck (BABIP, LOB%), and salary diversification (Gini), and we stratify according to two resource levels defined by SALn (Table 2).

**Table 2:** Logistic regression model. A LogWorth > 0.89 indicates statistical significance at the 0.05 level.

		*Below Average Resources*		*Above Average Resources*	
*Measure*	*Description*	*LogWorth*	*Coefficient*	*LogWorth*	*Coefficient*
FIP	Performance	14.42	-3.708	31.07	-5.036
LOB%	Luck	8.76	51.801	4.69	37.065
BABIP	Luck	1.36	-27.702	1.78	-38.653
Gini	Inequality	—-	—-	1.79	5.405
MCC	Model Success	0.611		0.641	
POD	Hit Rate	0.608		0.724	
FAR	False Alarm Ratio	0.244		0.205	

The model for above (below) average resources provides good discriminatory capacity, with a Mathews Correlation (MCC) coefficient [24] of 0.641 (0.611), probability of detection of 0.724 (0.608), and a false alarm ratio of 0.205 (0.244). For reference, these are standard measures of model performance for binary classifications. The MCC takes into account all four entries (true positives, false positives, true negatives, false negatives) for the binary classification contingency table and is a balanced measure even when class sizes are highly unequal. The probability of detection (POD or hit rate) simply measures the fraction of the true positives that were correctly classified, while the false alarm ratio (FAR) measures the fraction of the classified positives that did not occur.

**Fig 2:**
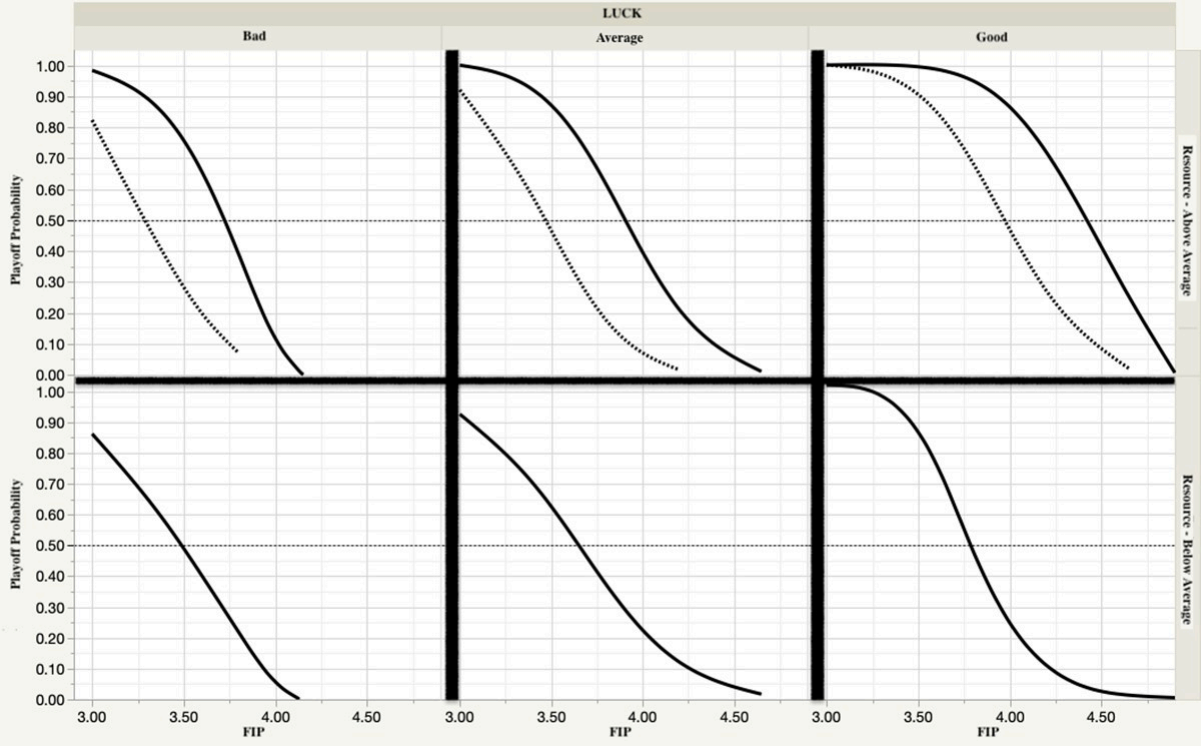
Playoff qualification chances—sensitivity to staff salary distribution, staff pitching performance (FIP) and luck (based on BABIP and LOB%). Top (bottom) panels are for teams with above (below) average pitching staff resources. For the above average resource panels, solid (dashed) lines indicate high (low) Gini coefficient.

Not surprisingly, the LogWorth [defined as -log10(*p*-value)], shows that FIP (i.e., realized performance) possesses the greatest diagnostic importance. Of particular interest is that the Gini coefficient is relevant only to the above average resource case. To visualize these effects, we plot the response of the model to a range of input values (Fig 2). Several conclusions are evident:

- teams with the resources that invest more in the “star” pitcher or pitchers on their staff at the expense of the remaining pitchers can increase their chances to make the playoffs;
- that investment will not pay off if the performance does not follow that implied by the salary;
- good or bad luck (BABIP) can have a non-negligible impact on the chances of both above and below average resource teams, but the former are more resilient to bad iuck

One might wonder why two above average resource pitching staffs with the same realized performance (as measured by FIP) would exhibit differing playoff qualification probabilities as a result of salary distribution. For example, suppose team A and team B each have staff FIP of 4.00 and equal amounts of good luck. But the management of team A devoted significant salary to two of its 5 starters, while team B, spread the same amount of salary dollars evenly across all of its starters. Our model predicts that team A will have a more than one-third higher chance of making the playoffs than team B. This result is a consequence of the relationship between “star” performance, salary and usage. Data for the period 1985–2016 indicate that the #1 pitcher will on average face over 70% more batters than the #5 pitcher on a staff. For two otherwise equal teams, to the extent that performance follows from allocated salary, this usage pattern alone will produce a few more staff wins over the course of a season than would otherwise be possible, thus enhancing the probability of qualifying for the playoffs. This effect is further enhanced in low run-scoring environments (see Eq 1).

## Discussion and conclusion

There exists a tension between risk and reward in all walks of life. Depending on many factors, a risky bet may provide value in one situation, while in other circumstances, such a bet would not be prudent. Here, we investigate whether the standard market investment advice–diversify a portfolio to reduce risk while working towards overall financial goals–makes sense in the context of the large financial bets that are placed on MLB starting pitchers.

Arguments for placing such a bet are: (1) the time horizon for a MLB team is one season, with the investment goal to make the playoffs (with considerable financial rewards therein); (2) the usage pattern for MLB pitchers is non-uniform, meaning that all else being equal, a high quality starter who is paid for an expected performance that he does deliver, will produce more wins than would be possible with the same amount of salary funds distributed more evenly across the pitching staff; (3) competitive market dynamics in MLB are such that a team will need to pay a high price to obtain a pitcher who has the potential (whether or not realized) to produce exceptional results; (4) a team in contention may need to add a relatively few amount of wins to guarantee a post-season position.

Arguments against placing such a bet are: (1) salary data show that expected and realized performance are only weakly correlated; (2) luck (both good and bad) can have a substantial influence on pitching outcomes, even with a sample as large as the 162 game MLB season; (3) the cost of adding a pitcher with high performance potential is high, so the cost of a bad bet can be damaging; (4) pitching injuries occur more frequently by a substantial margin than at any other MLB position and represent an additional risk; (5) given the variability in performance by both teams and individuals, it is difficult to accurately project how many additional wins may be needed to qualify for the post-season–a bet might not be needed, because it would not have changed the team’s playoff standing.

Given these arguments, it is not self-evident what the appropriate strategy should be. Motivated by this uncertainty, we performed a regression analysis, which supports the “all-in” strategy and thus runs contrary to standard financial investment advice. An important caveat is that this conclusion is connected to the financial resources of a team. One with limited funds is largely unable to compete in this marketplace and thus must depend primarily on good fortune, unexpected performance and market mispricing [25,26] to overcome their financial limitations. This can be more feasible when young, low-priced prospects are available to be brought to the team from the minor leagues, and subsequently perform at a high level. The investment required to obtain and nurture those talents, along with the added cost of the numerous development failures along the way, is beyond the scope of this analysis but would need to be considered. Accordingly a more complete analysis of MLB strategies would include investments across all aspects of the organization, beginning with the minor league system and continuing to major league roster construction, encompassing offense, position player defense, and the bullpen in addition to the starting pitching.

## Supporting information

S1 Dataset The dataset used in the study. (JMP)

S1 Metadata Descriptions of the dataset used in the study. (DOCX)
