# Supplementary material for: Does the principle of investment diversification apply to the starting pitching staffs of major league baseball teams?
Source: PLoS One. 2021 Jan 13;16(1):e0244941. doi: 10.1371/journal.pone.0244941 (PMC7806120; doi:10.1371/journal.pone.0244941)
Supplement: S1 Metadata — (DOCX) [file pone.0244941.s002.docx]

**Metadata**

The following is a list of metadata for the study on MLB starting pitcher staff salary diversification. The baseball performance data were obtained from baseballreference.com and fansgraphs.com, and the salary data were obtained from baseballreference.com. Gini indices were computed separately. Playoff qualification probabilities were calculated here. Each column is described below:

All the following are season summary statistics for each NBA team:

Season. The MLB year.

Team. Name of the MLB team.

LG. AL (American League) or NL (National League).

TM-ID. Three letter abbreviation for team.

ID. Combination of TM-ID and season.

Npit. Number of pitchers used during the season.

SAL($K). Starting pitcher salary allocation relative to the league average for that season (thousands of dollars). All starting pitching staff are based on the 5 pitchers who faced the most batters in the season.

LG-SAL.sd. League starting pitcher staff salary standard deviation (thousands of dollars).

SAL($K).norm. Standard normal starting pitcher staff salary.

Gini. Gini index for starting pitching staff salary.

W. Team wins in the regular season.

L. Team losses in the regular season.

GS. Games started in the regular season.

W162. Wins normalized for a 162 game regular season.

BABIP. Batting average on balls in play.

LOB%. Left on base percentage.

FIP. Fielder independent pitching on an earned run average scale.

TBF. Total batters faced by starting pithcing staff.

BF1-BF5. Batters faced by the each pitcher of the 5 man staff.

TM-GSC. Starting pitcher season weighted average game score (weighted by decisions of each pitcher).

W88. Binary classification – W162 ≥ 88 (1) or not (0).

GSC>52. Binary classification – TM-GSC ≥52 (1) or not (0).

Resource. Team starting staff salary above or below league average.

Lin[1] By Resource. Multiple logistic regression linear predictor function.

Prob[1] By Resource. Playoff qualification probability based on TM-GSC≥52.
